# Case Report: High efficacy of low-dose flecainide as an add-on therapy to a beta-blocker for treating a high burden of idiopathic ventricular arrhythmias in a juvenile athlete

**DOI:** 10.3389/fcvm.2025.1537078

**Published:** 2025-05-02

**Authors:** Georgios A. Christou, Konstantinos P. Letsas, Maria Konstandi, Maria A. Christou, Konstantinos A. Christou, Christos Kyriakopoulos, Eleftherios Vidalakis, Stelios Tigas, Dimitrios K. Christodoulou, Dimitrios N. Kiortsis

**Affiliations:** ^1^ Department of Radiology, Faculty of Medicine, University of Ioannina, Ioannina, Greece; ^2^ Atherothrombosis Research Centre, Faculty of Medicine, University of Ioannina, Ioannina, Greece; ^3^ Arrhythmia Unit, Onassis Cardiac Surgery Center, Athens, Greece; ^4^ Department of Pharmacology, Faculty of Medicine, University of Ioannina, Ioannina, Greece; ^5^ Department of Endocrinology, University Hospital of Ioannina, Ioannina, Greece; ^6^ Metropolitan General Hospital, Athens, Greece

**Keywords:** flecainide, sports cardiology, athlete, ventricular arrhythmias, case report

## Abstract

The detection of frequent premature ventricular contractions (PVCs) in an athlete represents one of the most important red flags during pre-participation screening. We report the case of a 6-year-old asymptomatic male athlete practicing basketball and sailing, who was examined for pre-participation screening. His resting electrocardiogram showed very frequent, isolated, monomorphic PVCs. The PVCs exhibited a left bundle branch block morphology with an inferior axis and R/S wave precordial transition in lead V3. The most likely origin of PVCs was considered the left ventricular outflow tract. Resting transthoracic echocardiography revealed reduced left ventricular systolic function, with an ejection fraction of 43%, indicating the possible existence of PVC-induced cardiomyopathy. We detected 43,149 isolated monomorphic PVCs (PVC burden: 40%) on 24-h ambulatory electrocardiographic monitoring. Initiation of treatment with atenolol 12.5 mg twice a day led to inadequate reduction of PVCs, with 29,452 isolated monomorphic PVCs (PVC burden: 29%) still observed on 24-h ambulatory electrocardiographic monitoring. After adding flecainide 25 mg twice daily to atenolol treatment, 24-h ambulatory electrocardiographic monitoring revealed complete resolution of ventricular arrhythmias, with no PVCs detected. Left ventricular systolic function recovered to normal. At 12 years of age, the athlete remained on combination therapy with atenolol and flecainide, continued participating in sports, and remained completely asymptomatic with normal cardiac examinations. The optimization of drug treatment was favored over catheter ablation since the athlete was a child and the probable origin of PVCs was the left ventricular outflow tract. This case report highlights that flecainide at a relatively low dose as an add-on therapy to a beta-blocker was highly effective and safe for treating high-burden PVCs originating from the ventricular outflow tract in a juvenile athlete.

## Introduction

Undoubtedly, the detection of frequent premature ventricular contractions (PVCs) in an athlete represents one of the most important red flags during pre-participation screening since PVCs not only potentially increase the risk of sudden cardiac death but may also signal the presence of an underlying cardiac disease (1, 2). Hence, an extensive diagnostic workup is essential for athletes presenting with frequent PVCs, and restriction from sports is recommended until the PVCs are effectively treated (1, 2). In some cases, invasive electrophysiological assessments may help reach a definitive diagnosis, especially when the clinical presentation is unclear (3, 4). According to the 2024 Heart Rhythm Society (HRS) guidelines for arrhythmias in athletes, treatment of PVCs in athletes without underlying cardiac disease is indicated when symptoms exist or a PVC-induced cardiomyopathy is diagnosed (1). Catheter ablation is recommended as a first-line option for treating PVCs in athletes due to its greater success rate and better tolerability compared to antiarrhythmic drug treatment (1). However, these recommendations may not be reliably extrapolated to juvenile athletes, particularly children, since the size of the ablation lesion is relatively larger compared to the child's heart and the risk of ablation-related complications is higher, including late lesion enlargement (5, 6). Furthermore, the level of recommendation for ablation of idiopathic PVCs is significantly lower for PVCs originating from the left ventricular outflow tract (LVOT) due to the lower success rate and higher risk of procedural complications (5). In this respect, the management of PVCs originating from the LVOT in juvenile athletes appears to be challenging enough to mandate alternative therapeutic strategies. Therefore, here, we report a case of a juvenile athlete with a very high burden of PVCs, probably originating from the LVOT, accompanied by PVC-induced cardiomyopathy. To our knowledge, no previous study has addressed this issue.

## Case description

A 6-year-old Caucasian male athlete, weighing 23 kg and practicing basketball and sailing, presented to a sports cardiology medical office for pre-participation screening. The athlete was asymptomatic and had no prior medical history. There was no family history of heart disease. He was not taking any dietary supplements or medications. His training regimen consisted of 2-h basketball training 3 days per week and 3-h sailing 2 days per week. Informed consent was obtained from the athlete and his parents. The study was performed in accordance with the Declaration of Helsinki. The study protocol was approved by the Ethics Committee of the University of Ioannina (13680/2024).

**Figure 1:**
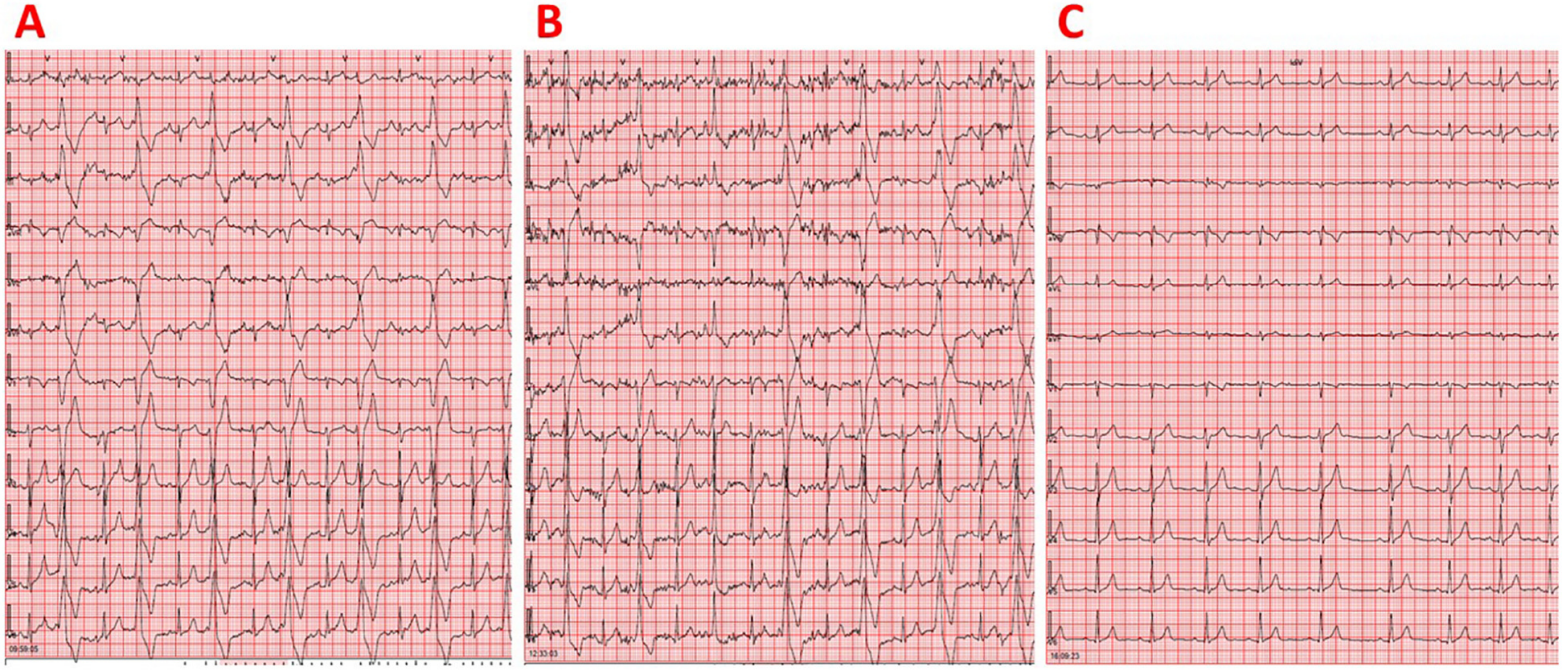
(**A**) Resting 12-lead electrocardiogram demonstrating sinus rhythm and very frequent isolated monomorphic PVCs in a juvenile athlete not taking any medications. The PVCs displayed a left bundle branch block morphology with an inferior axis and R/S wave precordial transition in lead V3. (**B**) Resting 12-lead electrocardiogram showing sinus rhythm and very frequent isolated monomorphic PVCs in a juvenile athlete taking atenolol 12.5 mg twice daily. The PVCs exhibited a left bundle branch block morphology with an inferior axis and R/S wave precordial transition in lead V3. (**C**) Resting 12-lead electrocardiogram demonstrating sinus rhythm without any PVCs in a juvenile athlete following combination therapy consisting of atenolol 12.5 mg twice daily and flecainide 25 mg twice daily. No QRS widening or QT interval prolongation was observed.

Physical examination revealed an irregular heartbeat on cardiac auscultation. The resting electrocardiogram showed a sinus rhythm with a heart rate of 81 bpm and very frequent isolated monomorphic PVCs (Figure 1A). The electrocardiographic waveforms corresponding to sinus beats were normal. The PVCs displayed a left bundle branch block morphology with an inferior axis and R/S wave precordial transition in lead V3. The most likely origin of the PVCs was considered the LVOT since the R-wave precordial transition occurred in the same lead (i.e., V3) as in sinus rhythm; additionally, the V2 transition ratio [i.e., the percentage R-wave of the PVC (R/R + S)PVC divided by the percentage R-wave in sinus rhythm (R/R + S)SR in V2] was >0.6, and the V2S/V3R index was <1.5 (7–10). Resting transthoracic echocardiography revealed reduced left ventricular systolic function, with an ejection fraction of 43%, profoundly indicating the existence of PVC-induced cardiomyopathy (Supplementary Table 1). We detected 43,149 isolated monomorphic PVCs on 24-h ambulatory (including an exercise session) electrocardiographic monitoring, with no pairs or episodes of ventricular tachycardia observed (Supplementary Table 2). The PVC burden, defined as the percentage of total beats that were PVCs, was 40%. After performing Spearman's correlation analysis, we found that the hourly PVC count was positively correlated with the corresponding mean heart rate (rho = 0.433, *p* = 0.044), implying the existence of a fast-heart rate-dependent PVC type (11). The athlete underwent a maximal exercise treadmill test using the MODBRUCE protocol. The test was stopped at 18:32 min (15.2 metabolic equivalents (METs)) due to exhaustion. Very frequent isolated monomorphic PVCs were detected before the start of the exercise and continued during exercise until the heart rate reached 130 bpm. Thereafter, for heart rates higher than 130 bpm, PVCs totally disappeared and appeared again during the recovery phase, but only when the heart rate fell below 130 bpm. No abnormal ST–T changes were noted during the exercise test or recovery phase. The athlete did not experience palpitations or syncope during the exercise test or recovery phase. Blood test results, including thyroid function tests and electrolytes, were normal. Moreover, cardiac magnetic resonance imaging revealed no evidence of myocardial edema, interstitial fibrosis, or replacement fibrosis.

**Figure 2:**
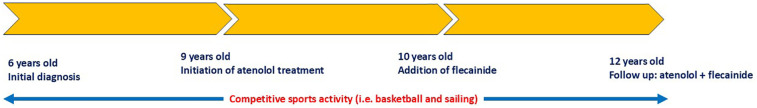
Timeline of events.

Initiation of treatment with atenolol 12.5 mg twice a day led to an inadequate reduction of PVCs since 24-h ambulatory electrocardiographic monitoring still recorded 29,452 isolated monomorphic PVCs, corresponding to a PVC burden of 29% (Figure 1B). A positive association was found between the hourly PVC count and the corresponding mean heart rate (rho = 0.934, *p* < 0.001), suggesting the existence of a fast-heart rate-dependent PVC type (8). After adding flecainide 25 mg twice daily to atenolol treatment, 24-h ambulatory electrocardiographic monitoring revealed complete resolution of ventricular arrhythmias, with no PVCs detected (Figure 1C, Supplementary Table 2). It should also be noted that there was no QRS widening or QT interval prolongation (12). Left ventricular systolic function recovered to normal after the initiation of flecainide (Supplementary Table 1). At 12 years of age (with a body weight of 58 kg, corresponding to a flecainide dose of 0.86 mg/kg/day), the athlete continued competitive sports activity (i.e., basketball and sailing) while remaining completely asymptomatic and maintaining normal cardiac examinations on combination therapy with atenolol and flecainide (Figure 2).

## Discussion

This case report demonstrates that flecainide at a relatively low dose as an add-on therapy to a beta-blocker completely suppressed PVCs originating from the ventricular outflow tract that were resistant to beta-blocker treatment in a juvenile athlete. The fact that the athlete was a child favored the optimization of medical treatment over catheter ablation because the size of an ablation-induced lesion is relatively large compared to a child's heart and the risk of ablation-related complications is higher in children than in adults (5). Furthermore, the probable LVOT origin of the PVCs could predict a lower success rate and higher risk of procedural complications of ablation, further reducing the indication for ablation (5).

Although the athlete was asymptomatic, the very high PVC burden associated with PVC-induced cardiomyopathy necessitated therapeutic interventions in accordance with the 2024 HRS guidelines for arrhythmias in athletes (1). Taking into account that the minimal threshold for PVC-induced cardiomyopathy has been found to be 10%, with a much higher risk when the PVC burden exceeds 20%, the remaining 29% PVC burden despite atenolol treatment prompted additional therapeutic interventions to further reduce the burden below 10% (1, 5, 13). Notably, atenolol treatment was ineffective in adequately reducing PVC burden despite the existence of a fast-heart rate-dependent PVC type, which has been reported to predict a successful decrease in PVC burden with beta-blocker therapy (11). Furthermore, athletes may find it difficult to tolerate uptitration of beta-blocker dosage due to the commonly encountered low heart rate in athletes, or it can even be detrimental to athletic performance (1).

The novelty of this study lies in the investigation of the efficacy and safety of low-dose flecainide treatment for a very high burden of PVCs originating from the ventricular outflow tract in a juvenile athlete. Although a few previous studies have reported the acceptable efficacy of flecainide in treating PVCs, the majority of them have not analyzed their data with respect to the site of PVC origin (14–17). The importance of this issue lies in the fact that the mechanism of outflow tract tachycardias involves triggered activity with delayed afterdepolarizations, which are mediated by cyclic adenosine monophosphate (cAMP) and downstream intracellular calcium release via the ryanodine receptor (8). Consistently, flecainide is an inhibitor of ryanodine receptor-mediated arrhythmogenic calcium release, possibly explaining its high efficacy in suppressing PVCs originating from the ventricular outflow tract in this case (18). Moreover, no previous study has evaluated the use of flecainide for treating PVCs originating from the outflow tract in juvenile individuals. One previous study by Hwang et al. reported the high efficacy of standard-dose flecainide for treating PVCs originating from the outflow tract in adult individuals (19). Unquestionably, pediatric patients present challenges in pharmacotherapy, not only in terms of efficacy but also safety, due to their modified pharmacokinetic and pharmacodynamic profiles compared to adults (20). Importantly, flecainide treatment was highly effective in eliminating PVCs even at a relatively low pediatric dose of 0.86 mg/kg/day, which was much lower than the maximum recommended pediatric dose of 8 mg/kg/day, thus minimizing the risk of proarrhythmia and other non-cardiac side effects, including hepatotoxicity (21, 22). Notably, the risk of drug interactions between flecainide and beta-blockers could be increased, including a potential synergistic effect leading to PR interval prolongation (23). To our knowledge, no previous study has evaluated the clinical effect of low-dose flecainide on ventricular arrhythmias in either pediatric or adult populations. Furthermore, the participation in sports of the individual in this case report poses an additional challenge in the management of his PVCs since athletic activity potentially enhances the risk of sudden cardiac death in the setting of ventricular arrhythmias (24). It should be acknowledged that a revision of the management strategy may be needed in the following years, including a trial of dose reduction or even discontinuation of flecainide treatment, as long-term spontaneous reduction of PVC burden has been previously found in a large proportion of children and adolescents (25). Discontinuation of sports activity was not deemed necessary in the athlete of this case report due to the previously reported neutral effect of detraining on PVC burden in juvenile athletes (25). Hence, in juvenile athletes with ventricular arrhythmias treated with antiarrhythmic drugs, a very careful consideration of all efficacy and safety issues is essential.

A limitation of this study was that the LVOT origin of PVCs was considered likely based only on electrocardiographic indices, without confirmation by 3D endocardial mapping. Moreover, it is also possible that the PVCs may have originated from an intramural or epicardial site, requiring further investigation. Furthermore, it should be acknowledged that complete resolution of PVCs was achieved with combination treatment involving atenolol and flecainide. Although the complete suppression of PVCs following the addition of flecainide to atenolol treatment implies that flecainide alone would be sufficient to eliminate the PVCs, the potential contribution of atenolol to this effect cannot be ruled out. Moreover, a maximal exercise stress test was not repeated after the introduction of flecainide. Even though such a test would have been useful to confirm that arrhythmias were well controlled during maximal effort and that the athlete maintained a normal chronotropic response, the athlete was instructed to perform a strenuous exercise session during repeated Holter monitoring, which provided comparable diagnostic information with exercise testing. In addition, although ablation was not considered a first choice in the athlete of this case due to the young age of the patient and the presumed LVOT origin of PVCs, it should be acknowledged that ablation represents a fundamental therapeutic tool for athletes with frequent PVCs. In fact, drug treatment may be ineffective, potentially associated with adverse events, or even intolerable, particularly in athletes, who commonly exhibit low heart rates (26).

In conclusion, flecainide at a relatively low dose as an add-on therapy to a beta-blocker was highly effective and safe for treating a high burden of PVCs originating from the ventricular outflow tract in a juvenile athlete. Therefore, low-dose flecainide represents a valuable option for treating ventricular arrhythmias originating from the outflow tract, particularly when catheter ablation is not recommended as a first-line treatment, such as in pediatric patients or in cases of LVOT-origin PVCs. Further studies are needed to evaluate the efficacy and safety of low-dose flecainide for treating ventricular arrhythmias originating from the LVOT not amenable to catheter ablation in athletes who are symptomatic or present with PVC-induced cardiomyopathy.

## Data Availability

The raw data supporting the conclusions of this article will be made available by the authors without undue reservation.
