# Supplementary material for: Case Report: High efficacy of low-dose flecainide as an add-on therapy to a beta-blocker for treating a high burden of idiopathic ventricular arrhythmias in a juvenile athlete
Source: Front Cardiovasc Med. 2025 May 2;12:1537078. doi: 10.3389/fcvm.2025.1537078 (PMC12081362; doi:10.3389/fcvm.2025.1537078)
Supplement: Supplementary file 1 [file Table1.docx]

**Supplementary Table 1.** Echocardiographic parameters at two time points: 1) before initiation of drug treatment and 2) during atenolol and flecainide treatment.

|  | **No drug treatment** | **Atenolol and flecainide treatment** |
| --- | --- | --- |
| **LVIVSd (cm)** | 0.4 | 0.6 |
| **LVPWTd (cm)** | 0.3 | 0.5 |
| **LVIDd (cm)** | 3.9 | 4.8 |
| **LVEDV (mL)** | 50 | 122 |
| **LVEF (%)** | 43 | 59 |
| **MVE (m/sec)** | 1.18 | 1.10 |
| **MVA (m/sec)** | 0.75 | 0.33 |
| **MVE/A** | 1.57 | 3.32 |
| **MVEa(s-l) (m/sec)** | 0.19 | 0.17 |
| **MVAa(s-l) (m/sec)** | 0.08 | 0.10 |
| **MVSa(s-l) (m/sec)** | 0.12 | 0.09 |
| **MVE/Ea(s-l)** | 6.2 | 6.4 |
| **LAD (cm)** | 2.2 | 3.0 |
| **TVSa (m/sec)** | 0.15 | 0.14 |
| **TAPSE (cm)** | 2.1 | 2.3 |

**Abbreviations.** LAD: left atrial diameter, LAV: left atrial end-systolic volume, LVEDV: left ventricular end-diastolic volume, LVEF: left ventricular ejection fraction, LVIDd: left ventricular end-diastolic internal diameter, LVIVSd: left ventricular interventricular septum thickness at end-diastole, LVPWTd: left ventricular posterior wall thickness at end-diastole, MVA: late diastolic transmitral flow velocity, MVA(s-l): average of septal and lateral late diastolic mitral annular velocity, MVE: early diastolic transmitral flow velocity, MVE/A: ratio of early to late diastolic transmitral flow velocity, MVEa(s-l): average of septal and lateral early diastolic mitral annular velocity, MVE/Ea(s-l): ratio of the early diastolic transmitral flow velocity to the average of septal and lateral early diastolic mitral annular velocity, MVSa(s-l): average of septal and lateral systolic mitral annular velocity, TAPSE: tricuspid annular plane systolic excursion, TVSa: systolic tricuspid annular velocity.
