# Supplementary material for: Case Report: High efficacy of low-dose flecainide as an add-on therapy to a beta-blocker for treating a high burden of idiopathic ventricular arrhythmias in a juvenile athlete
Source: Front Cardiovasc Med. 2025 May 2;12:1537078. doi: 10.3389/fcvm.2025.1537078 (PMC12081362; doi:10.3389/fcvm.2025.1537078)
Supplement: Supplementary file 2 [file Table2.docx]

**Supplementary Table 2.** Detailed results from Holter monitoring at three time points: 1) before initiation of drug treatment, 2) during atenolol treatment and 3) during atenolol and flecainide treatment.

|  | **No drug treatment** | **Atenolol treatment** | **Atenolol and flecainide treatment** |
| --- | --- | --- | --- |
| Minimum heart rate (bpm) | 42 | 43 | 42 |
| Maximum heart rate (bpm) | 136 | 126 | 158 |
| Average heart rate (bpm) | 75 | 71 | 61 |
| Total beats | 107,851 | 102,367 | 88,107 |
| PVCs | 43,149 | 29,452 | 0 |
| PVC burden (%) | 40 | 29 | 0 |
| Isolated PVCs | 43,149 | 29,452 | 0 |
| Pairs of PVCs | 0 | 0 | 0 |
| Ventricular tachycardia | 0 | 0 | 0 |
| Supraventricular extrasystoles | 0 | 0 | 0 |
| Pauses > 1.5 sec | 0 | 0 | 0 |

**Abbreviation.** bpm: beats per minute, PVCs: premature ventricular contractions.
